# Carbonating the household diet: a Pakistani tale

**DOI:** 10.1017/S1368980019004348

**Published:** 2020-06

**Authors:** Biplab K Datta, Muhammad Jami Husain

**Affiliations:** Global Noncommunicable Diseases Branch, Division of Global Health Protection, Center for Global Health, Centers for Disease Control and Prevention, Atlanta, GA, USA

**Keywords:** Carbonated beverages, Soft drinks, Pakistan, Expenditure elasticity

## Abstract

**Objective:**

Carbonated beverage consumption is associated with various adverse health conditions such as obesity, type 2 diabetes and CVD. Pakistan has a high burden of these health conditions. At the same time, the carbonated beverage industry is rapidly growing in Pakistan. In this context, we analyse the trends and socioeconomic factors associated with carbonated beverage consumption in Pakistan.

**Design:**

We use six waves of the cross-sectional household surveys from 2005–2006 to 2015–2016 to analyse carbonated beverage consumption. We examine the trends in carbonated beverage consumption-prevalence for different economic groups categorised by *per capita* household consumption quintiles. We estimate the expenditure elasticity of carbonated beverages for these groups using a two-stage budgeting system framework. We also construct concentration curves of carbonated beverage expenditure share to analyse the burden of expenditure across households of different economic status.

**Setting:**

Pakistan.

**Participants:**

Nationally representative sample of households in respective survey waves.

**Results:**

We find that the wealthier the household, the higher is the prevalence of carbonated beverage consumption, and the prevalence has increased for all household groups over time. From the expenditure elasticity analysis, we observe that carbonated beverages are becoming an essential part of food consumption particularly for wealthier households. And, lastly, poorer households are bearing a larger share of carbonated beverage expenditure in 2014–2016 than that in 2006–2008.

**Conclusion:**

Carbonated beverages are becoming an increasingly essential part of household food consumption in Pakistan. Concerns about added sugar intake can prompt consideration of public health approaches to reduce dietary causes of the disease burden in Pakistan.

Frequent intake of sugar-sweetened beverages (SSB) is a major risk factor associated with obesity, type 2 diabetes and CVD, kidney diseases, non-alcoholic liver disease, gout (a type of arthritis), tooth decay and cavities^(1–7)^. Obesity is a growing public health concern in Pakistan. With nearly one in every four adult (age >20) males and one in every three adult (age >20) females being overweight or obese, the country ranked ninth in the world in the number of individuals with obesity^(8)^. A large number of Pakistanis also suffer from diabetes mellitus, an epidemic that is emerging rapidly in recent years. A recent study reports 11 % pre-diabetes and 17 % type 2 diabetes prevalence in the population aged >20 years in Pakistan^(9)^. The burden of CVD in Pakistan is also high. CVD is the number one cause of death in Pakistan and accounted for 29 % of the total deaths in 2016^(10)^. SSB, being closely associated with these health conditions, have important policy relevance for population health in Pakistan.

SSB refer to any beverage with added sugar or other sweeteners, including soda, pop, cola, tonic, fruit punch, lemonade (and other ‘ades’), sweetened powdered drinks as well as sports and energy drinks. Carbonated beverages, commonly known as soda or soft drinks, are the most consumed type of SSB in Pakistan. Data from the Pakistan household income expenditure surveys^(11–16)^, used in the current study, show that carbonated beverages constitute nearly 60–70 % of the household-level consumption of non-alcoholic beverages (see online supplementary material, Supplemental Table 1 for details). Carbonated beverage industry is also an important and thriving manufacturing sector, which is a major source of revenue for the Government of Pakistan in the form of federal excise duty and domestic sales tax^(17)^. Despite being a sizable economic sector on one side and an important public health issue on the other side, there is lack of analyses on the trends and other socioeconomic aspects of carbonated beverage consumption in Pakistan. In this paper, we analyse the household-level consumption of carbonated beverages over a decade (i.e., 2006–2016) to better understand the socioeconomic factors associated with carbonated beverage consumption, which could inform policy-makers to promote health awareness and adopt other preventive measures.

Several studies show the increasing global trends and regional heterogeneity in SSB consumption in recent years^(18,19)^. Several other studies provide country-specific elasticity estimates of SSB^(20–23)^. Demand for cola carbonates is rapidly growing in Pakistan, escalating the battle for market share among the major global cola producers^(24)^; yet, no in-depth analysis of household-level carbonated beverage consumption is available for Pakistan. The production of carbonated beverages in Pakistan has been increasing gradually over time, accompanied by a declining trend in real price (Fig. 1). Together it contributed in greater affordability of carbonated beverages in Pakistan. Though the real price shows some upward trend in recent years (2015–2016), it is still way below the price in 2006. A study showed that SSB became more affordable in Pakistan from 1990 to 2016 as the real price of SSB decreased by $US 3·84 (constant 2010 dollars) during that period^(25)^. In this context, studying the socioeconomic aspects of carbonated beverages consumption in Pakistan is crucial for public health interventions to prevent and control obesity, type 2 diabetes, CVD and other adverse health conditions.

**Fig. 1:**
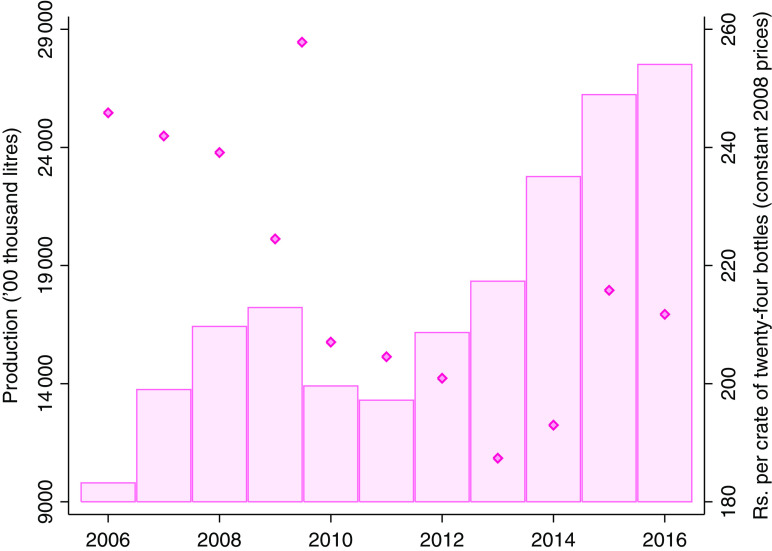
Price and production of carbonated beverages in Pakistan. Production data are obtained from quantum index of large-scale manufacturing industries reported by the Pakistan Bureau of Statistics, and from the Industrial Commodity Statistics Database of the United Nations Statistics Division. Nominal price data are obtained from the Pakistan Statistical Year Book. Prices are of crate of 24 bottles of Coca Cola and 7 Up. Constant prices are obtained using annual consumer price index (CPI) measures. 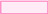, production; 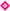, price

Using data from the household income expenditure surveys, the current study aimed to, first, analyse the trend in carbonated beverage consumption across households of different economic status over a decade (from 2006 to 2016); second, estimate the expenditure elasticity of carbonated beverages for different income groups; third, analyse the evolution of expenditure elasticities over time; and fourth, analyse the carbonated beverage expenditure share burden across income groups over time.

## Methods

### Data

We use data from the 2005–2006, 2007–2008, 2010–2011, 2011–2012, 2013–2014 and 2015–2016 waves of the Pakistan Household Integrated Economic Survey (HIES)^(11–16)^. HIES is a stratified two-stage nationally representative survey that covers a large number of rural and urban households from twenty-seven administrative divisions of Pakistan in four provinces^(16)^. HIES provides detailed information on household food and non-food consumption, including fortnightly (2 weeks) consumption (quantity and expenditure) of carbonated beverages. According to the Pakistani standards, carbonated beverages are defined as non-alcoholic beverages that contain dissolved carbon dioxide with addition of mineral salts, sugar and/or other sweetener, flavours, colours and other food additives^(26)^. We derived households’ monthly carbonated beverage consumption using the fortnightly expenditure data reported in HIES.

### Consumption trend

We examine the trends in household-level prevalence of carbonated beverage consumption from 2006 to 2016. To smooth out short-term fluctuations, we pooled two consecutive HIES cohorts of 2005–2006 and 2007–2008, 2010–2011 and 2011–2012, and 2013–2014 and 2015–2016 into three time periods – 2006–2008, 2011–2012, 2014–2016 – and compared prevalence across the time periods. We analysed the consumption trend by household’s economic status. We categorised households by monthly household expenditure *per capita* quintiles and compared prevalence across quintiles. We then compared mean expenditure share of carbonated beverages as a percentage of monthly food expenditure across quintiles and time periods. We also examined the trends in mean consumption quantity *per capita* and the mean expenditure per litre.

### Expenditure elasticity estimation

We used a two-stage budgeting system methodology^(27)^ for estimating expenditure elasticity of carbonated beverages in Pakistan. In the first stage, we categorised household consumptions in several broad groups (e.g., food, clothing, housing, etc.) and estimated expenditure elasticity of the broad food category. In the second stage, we estimated expenditure elasticity of carbonated beverages within the broad food category. The idea behind the two-stage budgeting is that households first decide how much to spend on each broad category and then allocate spending for within-category consumption in the second stage. A similar method was applied by Menezes *et al*.^(28)^ for estimating elasticities for food products in Brazil.

Household expenditures in HIES are categorised into eleven broad categories, which are food, tobacco, clothing, housing, education, fuel and utilities, personal care, transportation, recreation, medical spending, and miscellaneous. Food items are categorised in sixteen sub-categories, which are dairy, meat and fish, fresh fruits, dried fruits and nuts, vegetables, condiments and spices, sugar, readymade food, baked and fried products, cereals, legumes, edible oil and fats, tea and coffee, carbonated beverages, other non-alcoholic beverages, and miscellaneous food items. Hence, we estimated a system of eleven equations in the first stage, and another system of sixteen equations in the second stage. The miscellaneous category in both first and second stage was omitted to satisfy the summation restriction (i.e., category expenditure shares add up to 1). Like the consumption trend analysis, we estimated the equations for three time periods by pooling two rounds of HIES in each period.

We estimated a Quadratic Almost Ideal Demand System (QAIDS) proposed by Banks *et al.* ^(29)^ using the seemingly unrelated regression (SUR) model. The empirical analysis was conducted using Stata 13.1. We assumed that commodities are weakly separable in household’s utility function and estimated the following equation in the first stage:
(1)

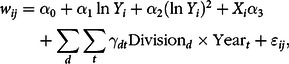

where *w*_*ij*_ is the expenditure share of *j*th category of household *i*; ln *Y*_*i*_ is the log of total monthly expenditure (constant 2008 Rs.) of non-durable commodities; ***X***_***i***_ is a vector of household-specific sociodemographic characteristics; 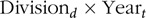 is the division–year fixed effect; and *ϵ*_*ij*_ is the idiosyncratic error term. Since we do not observe prices faced by the households, following the lead of Deaton^(30)^, we utilised spatial variation in commodity prices across administrative divisions over time. We assumed that all households located in division *d* in year *t* faced similar prices for commodity *j*. Therefore, along with other division-level unobserved factors (e.g., food habits, social norms, etc.), division–year fixed effects accounted for prices in the regression. A squared term of log expenditure, (ln *Y*_*i*_)^2^, was added in the model to allow for commodities deemed a necessity or luxury depending on household *i*’s expenditure level. The vector ***X***_***i***_ includes controls for household’s urban or rural residence, dwelling type, occupancy status, source of drinking water, whether the household has gas connection, type of toilet used, type of sewerage system, whether the household receives remittance from abroad, whether the household receives government cash transfer, whether the household produces food crops, whether the household owns poultry, whether the household owns livestock, whether the household takes loan to finance consumption expenditure during the survey period, share of children aged under 5, share of elderly (age >65), whether the household has school-going (6–14) children, whether the household has reproductive-age female (15–49), share of adult male (age >18), household size and household head’s education. These covariates were included in the model to account for household-specific behaviours that may impact certain commodity consumption, and to obtain more precise estimates of *α*_*1*_ and *α*_*2*_. The coefficient estimates of *α*_*1*_ and *α*_*2*_ jointly determine, along with the level of expenditure *Y*, the marginal effect of log of total household expenditure on household’s expenditure share of a certain broad category. In the context of our study, the coefficients alone were not meaningfully interpretable, rather they were later used to estimate expenditure elasticities.

Expenditure elasticity describes how responsive the households are in adjusting consumption of a certain commodity, following any changes in household income or expenditure. The higher the expenditure elasticity, the larger is the responsiveness of quantity demanded to any change in expenditure or income. Higher (>1) expenditure elasticity means the commodity is a luxury item in household’s consumption basket. An increase in expenditure in such case is associated with more than a proportionate increase in quantity demanded. Conversely, a decrease in expenditure elasticity, therefore, refers to relatively less responsiveness of quantity demanded, meaning consumers do not change their consumption by much following any changes in expenditure. An expenditure elasticity <1 means the commodity is a necessity in household’s consumption basket.

Using the estimates of *α*_*1*_ and *α*_*2*_ of the respective period, we estimated expenditure elasticities for each quintile for each of the three periods. The expenditure elasticity of the *j*th commodity for the *q*th quintile, *η*_*jq*_, was calculated using equation (2):
(2)

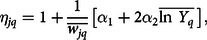

where 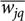 is the *j*th commodity’s mean expenditure share at the *q*th quintile, and 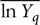 is the mean log monthly expenditure at the *q*th quintile. We assumed that households do not move across quintiles during the two survey cohort periods. We also calculated *η*_*j*_ for all households, where *w*_*j*_ and ln *Y* are averaged over the full sample for respective periods.

A similar specification like equation 1 was estimated in the second stage, where *w*_*ij*_ was replaced with *f*_*ik*_, the food expenditure share of the *k*th food category; and *Y*_*i*_ was replaced with *F*_*i*_, the monthly food expenditure. The expenditure elasticity of the *k*th food category of *q*th quintile, *η*_*(j)kq*_, was then estimated using equation (2), respectively replacing *w* with *f* and *Y* with *F*. Following Carpentier and Guyomard^(31)^, the total expenditure elasticity of the *k*th food category, *E*_*k*_, was then calculated using the following formula:
(3)

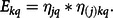




In the ‘Results’ section, we report the expenditure elasticity of food, *η*_Food_, from the first stage estimation; within food category expenditure elasticity of carbonated beverages, *η*_(Food)*CB*_, from second stage estimation; and the total expenditure elasticity of carbonated beverage, *E*_*CB*_, calculated using the estimates from the first and second stage elasticities. We compared the total expenditure elasticities across three time periods and across quintiles.

### Expenditure share burden

Finally, we examined how the expenditure share of carbonated beverage consumption was spread across households of different economic status (e.g., poor *v.* rich) over the periods. We organised households by *per capita* consumption percentile in each period and calculated the share of carbonated beverage spending for each percentile. We then generated concentration curves by plotting the cumulative expenditure share against household consumption *per capita* percentile for each period. A point (*p,s*) on the concentration curve of a period, where *p* and *s* refer to the coordinates of horizontal and vertical axes, respectively, can be interpreted as *s* % of the carbonated beverage expenditure share in that period being borne by the bottom *p* % of all households. We compared the concentration curves of 2006–2008, 2011–2012 and 2014–2016 to examine how the burden of carbonated beverage expenditure changed over time.

## Results

HIES provided household-level consumption information of 15 453, 15 512, 16 341, 15 807, 17 991 and 24 238 households in respective survey waves. The household-level prevalence of carbonated beverage consumption is reported in Table 1. For each period, it was evident that higher the quintile (i.e., wealthier the household), the higher the consumption prevalence. The difference in prevalence between the top (fifth) and the bottom (first) quintile was >40 percentage points. The prevalence gradually increased over time for every household group. During 2006–2008, around 26 % of the households in Pakistan consumed carbonated beverages, which increased by >10 percentage points in 2014–2016. The increase in prevalence was the highest for the fourth quintile (13·4 percentage points) and lowest for the first quintile (7·8 percentage points).

**Table 1:** Household-level carbonated beverage consumption prevalence and expenditure share

	Proportion of households consuming carbonated beverages (%)						Average expenditure* as a share of food expenditure (%)					
	2006–2008		2011–2012		2014–2016		2006–2008		2011–2012		2014–2016	
	%	95 % CI	%	95 % CI	%	95 % CI	%	95 % CI	%	95 % CI	%	95 % CI
Quintile 1	9·24	8·54, 9·94	14·25	13·39, 15·10	17·08	16·23, 17·93	0·12	0·11, 0·13	0·16	0·15, 0·17	0·10	0·10, 0·11
Quintile 2	16·2	15·28, 17·11	21·95	20·95, 22·94	26·97	26·02, 27·93	0·19	0·18, 0·20	0·24	0·23, 0·25	0·16	0·15, 0·17
Quintile 3	23·51	22·45, 24·58	28·99	27·89, 30·09	35·33	34·30, 36·35	0·28	0·27, 0·30	0·31	0·30, 0·32	0·21	0·20, 0·22
Quintile 4	31·29	30·08, 32·50	39·77	38·54, 41·00	44·69	43·64, 45·73	0·36	0·35, 0·38	0·42	0·40, 0·44	0·26	0·25, 0·27
Quintile 5	51·25	49·97, 52·52	59·26	58·04, 60·48	59·71	58·71, 60·70	0·66	0·63, 0·68	0·64	0·62, 0·66	0·32	0·31, 0·33
All	25·79	25·29, 26·28	32·55	32·04, 33·07	37·75	37·29, 38·21	0·32	0·31, 0·33	0·35	0·34, 0·36	0·22	0·21, 0·22

* Calculation of average expenditure share includes both user and non-user households.

The average carbonated beverage expenditure share also demonstrated a similar pattern that higher the quintile, the higher the expenditure share (Table 1). However, unlike the prevalence, the average expenditure share decreased from 2006–2008 to 2014–2016. In Table 2, we further explore this issue by examining trends in consumption *per capita* and average unit expenditure. We found that average *per capita* monthly carbonated beverage consumption increased from 0·85 litre in 2006–2008 to 1·03 litre in 2014–2016, whereas average expenditure per litre decreased from Rs. 39 to Rs. 31·5 during the same period. Together this suggests that the decrease in average expenditure share over time was due to a decrease in average unit expenditure and not because of decrease in quantity consumed.

**Table 2:** *Per capita* consumption and average unit expenditure of households consuming carbonated beverages

	Consumption *per capita* * (l)						Average unit expenditure per litre† (constant 2008 Rs.)					
	2006–2008		2011–2012		2014–2016		2006–2008		2011–2012		2014–2016	
	%	95 % CI	%	95 % CI	%	95 % CI	%	95 % CI	%	95 % CI	%	95 % CI
Quintile 1	0·35	0·33, 0·36	0·34	0·33, 0·35	0·48	0·46, 0·50	40·15	39·47, 40·84	45·68	44·19, 47·16	32·15	31·54, 32·76
Quintile 2	0·43	0·41, 0·44	0·46	0·44, 0·47	0·61	0·59, 0·62	39·44	38·85, 40·03	43·86	42·75, 44·96	31·95	31·53, 32·37
Quintile 3	0·54	0·52, 0·56	0·58	0·56, 0·59	0·76	0·74, 0·78	39·19	38·71, 39·68	42·49	41·54, 43·43	31·53	31·19, 31·87
Quintile 4	0·71	0·62, 0·80	0·72	0·70, 0·74	0·95	0·93, 0·97	38·42	38·02, 38·82	41·07	40·28, 41·86	31·34	31·04, 31·65
Quintile 5	1·31	1·26, 1·37	1·28	1·24, 1·31	1·54	1·50, 1·57	38·79	38·47, 39·11	38·68	38·11, 39·26	31·11	30·87, 31·35
All	0·85	0·82, 0·88	0·82	0·81, 0·84	1·03	1·02, 1·05	38·97	38·77, 39·17	41·27	40·89, 41·66	31·45	31·30, 31·60

* Consumption *per capita* was calculated by dividing the monthly consumption quantity (in litres) by household size.

† Average unit expenditure was calculated by dividing monthly consumption expenditure by monthly consumption quantity. Constant Rs. were obtained using general Consumer Price Index measures.

The expenditure elasticity results are presented in Table 3. The higher the absolute values of elasticity, the greater the responsiveness of households to an increase or decrease in household expenditure. It showed that a 1 % increase in household expenditure is associated with, respectively, 1·66 and 1·36 % increase in expenditure for carbonated beverages in Pakistan in 2006–2008 and 2014–2016. Decline in elasticity values meant that carbonated beverages have become relatively an integral part of household consumption over the years. We found that the expenditure elasticities of carbonated beverages were positive for all quintiles, and the higher the quintile, the lower the expenditure elasticity at every period. This meant that an increase in household expenditure (income) at the bottom quintile would result in a relatively larger increase in quantity demanded of carbonated beverages, compared with that at the upper quintiles. This suggests that carbonated beverage consumption in Pakistan is strongly associated with households’ economic status and becomes a relatively necessary part of households’ diet as expenditure (income) increases.

**Table 3:** Expenditure elasticity estimates

	Expenditure elasticity of food (*η*_Food_)						Within-food category expenditure elasticity of carbonated beverages (*η*_(Food)*CB*_)								
	2006–2008		2011–2012		2014–2016		2006–2008		2011–2012		2014–2016		Total expenditure elasticity (*E*_*CB*_)*		
	%	95 % CI†	%	95 % CI†	%	95 % CI†	%	95 % CI†	%	95 % CI†	%	95 % CI†	2006–2008	2011–2012	2014–2016
Q1	0·88	0·87, 0·88	0·89	0·88, 0·90	0·87	0·86, 0·87	3·70	3·52, 3·87	2·59	2·42, 2·76	2·93	2·82, 3·03	3·24	2·31	2·53
Q2	0·86	0·85, 0·86	0·87	0·86, 0·88	0·85	0·84, 0·85	2·67	2·56, 2·77	2·01	1·91, 2·11	2·02	1·96, 2·08	2·28	1·75	1·72
Q3	0·84	0·83, 0·85	0·85	0·85, 0·86	0·83	0·83, 0·84	2·12	2·05, 2·18	1·76	1·69, 1·84	1·70	1·66, 1·75	1·78	1·50	1·42
Q4	0·82	0·81, 0·82	0·83	0·82, 0·84	0·81	0·81, 0·82	1·87	1·82, 1·92	1·55	1·5, 1·61	1·47	1·44, 1·51	1·53	1·29	1·20
Q5	0·72	0·71, 0·73	0·75	0·74, 0·76	0·76	0·75, 0·76	1·47	1·44, 1·5	1·35	1·31, 1·39	1·26	1·23, 1·29	1·06	1·01	0·95
All	0·83	0·83, 0·84		0·84, 0·85	0·83	0·82, 0·83	2·00	1·94, 2·06	1·68	1·61, 1·74	1·65	1·61, 1·7	1·66	1·42	1·36

* *E*_*CB*_ is the product of *η*_*F*_ and *η*_*(F)CB*_.

† 95 % CI were calculated using the delta method.

The expenditure elasticity of the broad food category does not change much over time (for all quintiles). The within-food category expenditure elasticities of carbonated beverages, on the other hand, showed changes over time for all household quintiles. The within-food category elasticity changes were, therefore, the main driver of changes in estimated total elasticities. The total expenditure elasticity of carbonated beverages decreased by 0·30 percentage points from 2006–2008 to 2014–2016. The decrease occurred for all quintiles and was the highest for the first quintile (0·71 percentage points) and lowest for the fifth quintile (0·11 percentage points). The expenditure elasticity for the fifth quintile became <1 in 2014–2016, suggesting that carbonated beverage became a necessary commodity for wealthier households in Pakistan. We also estimated the elasticities without controlling for household characteristics, and the unadjusted estimates were found very similar to the adjusted estimates presented in Table 3 (see online supplementary material, Supplemental Table 2 for unadjusted elasticity estimates).

**Fig. 2:**
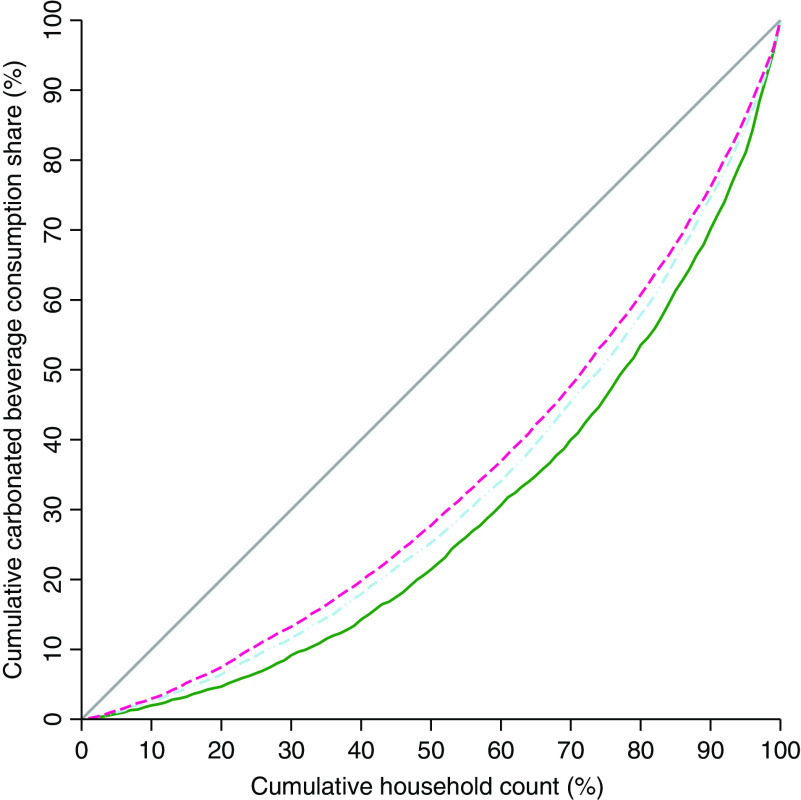
Concentration curve of expenditure share. Cumulative household count is based on household’s economic status in ascending order. *x*% in the horizontal axis refers to poorest *x*% households. 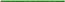, 2006–2008; 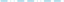, 2011–2012; 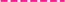, 2014–2016; 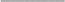, equality line

Finally, the analysis of expenditure burden share is presented in Fig. 2. The distant the concentration curve from the equality line, the lesser the bottom *x*% of the households’ share in carbonated beverage consumption. We found that the concentration curve has gradually shifted upward over time, that is, got closer to the equality line, meaning the bottom *x*% of the households are bearing a greater share of carbonated beverage expenditure in 2014–2016 than that in 2006–2008. The bottom 30 % households bore 9·2 % of the expenditure share in 2006–2008, which increased to 11·6 % in 2011–2012 and further increased to 13·2 % in 2014–2016. For the bottom 50 % households, it increased from 21·4 to 25·3 to 27·7 % during the same periods. Hence, not only more poorer households were consuming carbonated beverages in Pakistan in recent years, the expenditure share burden of carbonated beverage spending of poorer households had increased as well. This shift in burden over time may have detrimental consequences on poorer households, who otherwise could have spent the money on other food items.

## Discussion

Our analyses provide evidence on two important aspects of household-level prevalence of carbonated beverage consumption in Pakistan. First, we found the prevalence had a strong association with households’ economic status. The wealthier the household, the higher the prevalence of carbonated beverage consumption. And second, the prevalence had increased for all household groups (e.g., poor, middle class, rich) over time. Our analyses also inform two vital socioeconomic observations related to carbonated beverage consumption. First, carbonated beverages have gradually become an integral part of Pakistani diet, and particularly for the rich households, it became an essential consumption good. And second, poorer households bore a larger share of carbonated beverage expenditure in 2015–2016 than a decade ago. These results have important socioeconomic and public health considerations.

Over the years, expenditure elasticities of carbonated beverages in Pakistan have been gradually declining for all income groups. For example, at the bottom quintile, a 1 % increase in expenditure in 2006–2008 was associated with 3·24 % increase in quantity demanded, which decreased to 2·53 % in 2014–2016 – a relatively lower degree of response, meaning consumers changed consumption by a lower amount than that in 2006–2008 due to the same change in real expenditure (income). This suggests that carbonated beverages have become a relatively essential good in Pakistan over time. This is a serious public health concern since sugar-sweetened carbonated beverage consumption is associated with various adverse health conditions.

Though several studies have estimated the price elasticities of SSB^(21–23)^, very few provided estimates of expenditure elasticities. One study on soft drinks consumption in Guatemala reported an expenditure elasticity estimate of 0·99^(20)^. This estimate is lower than our overall expenditure elasticity estimate of 1·36 in 2014–2016, but very close to that (0·95) for the wealthiest households during the same period. Future research in this area, using data from various other countries, will facilitate cross-country comparison of expenditure elasticities, and will enhance our understanding of this issue in the global context.

The finding that poorer households are bearing a larger share of carbonated beverages in 2014–2016 than in 2006–2008 raises equity concerns. Since the expenditure elasticities of food remained unchanged over time, poorer households were likely to substitute other food consumptions with carbonated beverages. If they substituted carbonated beverages with some nutritious food items like milk, then that could adversely affect health outcomes, particularly for the children. HIES data show that expenditure on dairy products as a percentage of total food expenditure for the bottom quintile decreased from 17 % in 2006–2008 to 14 % in 2014–2016, indicating a possibility of substitution. The increase in consumption prevalence for the poorer households may also increase the risk of associated adverse health conditions. Studies showed that medication expenditures of major non-communicable diseases (e.g., blood pressure, diabetes) are strongly associated with incurring catastrophic health expenditures in Pakistan^(32)^. Carbonated beverage-attributable health conditions, thus, could further aggravate the poor households’ quality of life.

Lichtenstein^(33)^ argues that enough evidence on the adverse effects of SSB has already been documented, and now it is time to focus on understanding the drivers of SSB consumption, so that efforts can be made to fix the bigger public health problem without any further delay. Our study has great relevance to this view as we assessed the socioeconomic factors associated with household-level carbonated beverage consumption in Pakistan. Our expenditure elasticity estimations for different periods and for different economic groups portrayed a reliable depiction of the evolution of carbonated beverage consumption in Pakistan by controlling for a rich set of socioeconomic variables. Thus, our findings provide insights for effective public health policy interventions.

One limitation of our study is the lack of actual price data, which we proxied by assuming that households residing within an administrative division in the survey year faced similar product pricing. Though we obtained unit values by dividing expenditure amount by quantity consumed, we did not treat these values as prices because of endogeneity concerns emanating from measurement errors and ignoring quality variations. Knowing the actual price data could deliver more precise estimates. Second, HIES does not provide information on different types and brands of carbonated beverages consumed by the households. Some of the carbonated beverages consumed by households may be unsweetened and diet or low-calorie beverages, which we could not distinguish in our analysis. Third, we analysed the household-level consumption of carbonated beverages and could not derive the individual-level consumption. Behavioural determinants of carbonated beverage consumption could be useful in designing targeted awareness campaigns and prevention programmes for certain demographic groups (e.g., teenagers). However, our data do not permit individual-level or within-household consumption of carbonated beverages. Future research in addressing this gap will be very helpful.

## Conclusion

Our analyses generated evidence on the trends and economic group-specific patterns of carbonated beverage consumption. These findings could be utilised as background information to initiate efforts for reducing carbonated beverage consumption. Efforts may include promoting behavioural changes through awareness campaigns or influencing consumption through fiscal interventions like taxation. Imposing tax on sugary beverages may lead to a reduction in consumption and might also result in healthcare cost savings^(34,35)^. Though Pakistan has a federal excise duty and general sales tax on carbonated beverages, the existing tax rates may not be adequate to curb carbonated beverage consumption. We observed a declining trend in real prices (measured by average per unit expenditure) of carbonated beverages over time, which may escalate consumption prevalence. Policy-makers in Pakistan may, therefore, need to re-assess the tax structure associated with carbonated beverages to decrease affordability and, thereby, reduce consumption.

## Supplementary material

For supplementary material accompanying this paper visit https://doi.org/10.1017/S1368980019004348. click here to view supplementary material
