# Supplementary material for: Carbonating the household diet: a Pakistani tale
Source: Public Health Nutr. 2020 Mar 20;23(9):1629–37. doi: 10.1017/S1368980019004348 (PMC7196008; doi:10.1017/S1368980019004348)
Supplement: Supplementary file 1 [file S1368980019004348sup001.docx]

**Appendix**

**Table A1** Share of carbonated beverages in households’ nonalcoholic beverage consumption

| Survey* | Households’ total monthly expenditure† on nonalcoholic beverages‡ (million Rs.) | Households’ total monthly expenditure† on carbonated beverages  (million Rs.) | Share of carbonated beverages in households’ total expenditure of nonalcoholic beverages  (%) |
| --- | --- | --- | --- |
|  |  |  |  |
| 2005-06 | 464.80 | 334.00 | 71.86 |
| 2007-08 | 650.80 | 459.00 | 70.53 |
| 2010-11 | 1135.00 | 843.00 | 74.27 |
| 2011-12 | 964.50 | 759.00 | 78.69 |
| 2013-14 | 1992.60 | 1430.00 | 71.77 |
| 2015-16 | 2739.90 | 1690.00 | 61.68 |
|  |  |  |  |

*Pakistan Household Integrated Economic Survey, various waves. Pakistan Bureau of Statistics, Islamabad: Government of Pakistan.

†Total monthly expenditure is the aggregate average monthly expenditure of all households and calculated using wave specific complex survey weights.

‡Nonalcoholic beverages include carbonated beverages, squashes and syrups, fresh and packed fruit juices, and mineral water.

**Table A2** Expenditure elasticity estimates without controlling for household characteristics

|  | Expenditure Elasticity of Food (η_Food_) | | | Within Food Category Expenditure Elasticity of Carbonated Beverages (η_(Food)CB_) | | | Total Expenditure Elasticity  (E_CB_)† | | |
| --- | --- | --- | --- | --- | --- | --- | --- | --- | --- |
|  | **2006-2008** | **2011-2012** | **2014-2016** | **2006-**  **2008** | **2011-**  **2012** | **2014-**  **2016** | **2006-2008** | **2011-2012** | **2014-2016** |
| Q1 | 0.90 | 0.92 | 0.89 | 3.08 | 2.54 | 2.55 | 2.76 | 2.34 | 2.26 |
|  | (0.89, 0.90)* | (0.92, 0.93) | (0.88, 0.89) | (2.94, 3.21) | (2.44, 2.64) | (2.46, 2.63) |  |  |  |
| Q2 | 0.87 | 0.89 | 0.86 | 2.34 | 1.98 | 1.82 | 2.03 | 1.76 | 1.57 |
|  | (0.86, 0.87) | (0.88, 0.89) | (0.86, 0.87) | (2.27, 2.42) | (1.93, 2.04) | (1.77, 1.86) |  |  |  |
| Q3 | 0.84 | 0.87 | 0.85 | 1.92 | 1.74 | 1.56 | 1.62 | 1.51 | 1.32 |
|  | (0.84, 0.85) | (0.86, 0.87) | (0.84, 0.85) | (1.87, 1.97) | (1.70, 1.79) | (1.52, 1.59) |  |  |  |
| Q4 | 0.81 | 0.84 | 0.82 | 1.73 | 1.54 | 1.37 | 1.41 | 1.29 | 1.12 |
|  | (0.81, 0.82) | (0.83, 0.84) | (0.82, 0.82) | (1.69, 1.77) | (1.51, 1.57) | (1.35, 1.40) |  |  |  |
| Q5 | 0.68 | 0.72 | 0.74 | 1.42 | 1.34 | 1.20 | 0.96 | 0.97 | 0.89 |
|  | (0.67, 0.69) | (0.72, 0.73) | (0.73, 0.75) | (1.39, 1.44) | (1.32, 1.37) | (1.18, 1.22) |  |  |  |
| All | 0.83 | 0.86 | 0.83 | 1.82 | 1.66 | 1.52 | 1.52 | 1.42 | 1.26 |
|  | (0.83, 0.84) | (0.85, 0.86) | (0.83, 0.84) | (1.78, 1.87) | (1.62, 1.70) | (1.48, 1.55) | 2.76 | 2.34 | 2.26 |

*95% confidence intervals, calculated using the delta method, are in parenthesis.

†E_CB_ is the product of η_F_ and η_(F)CB_.
